# Clinicopathological characteristics of localized prostate cancer in younger men aged ≤ 50 years treated with radical prostatectomy in the PSA era: A systematic review and meta‐analysis

**DOI:** 10.1002/cam4.3320

**Published:** 2020-07-22

**Authors:** Yu Zheng, Sharron X. Lin, Shulin Wu, Douglas M. Dahl, Michael L. Blute, Wei‐De Zhong, Xing Zhou, Chin‐Lee Wu

**Affiliations:** ^1^ Department of Urology The Second Affiliated Hospital of Guangzhou Medical University Guangzhou Medical University Guangzhou China; ^2^ Department of Urology Massachusetts General Hospital Harvard Medical School Boston MA USA; ^3^ Department of Pathology Massachusetts General Hospital Harvard Medical School Boston MA USA; ^4^ Department of Urology Guangdong Key Laboratory of Clinical Molecular Medicine and Diagnostics Guangzhou First People's Hospital Guangzhou Medical University Guangzhou China

**Keywords:** active surveillance, meta‐analysis, prognosis, prostate cancer, radical prostatectomy, systematic review, younger age

## Abstract

**Objectives:**

With the rapid increase in younger age prostate cancer (PCa) patients, the impact of younger age on decision‐making for PCa treatment needs to be revaluated in the new era.

**Materials and Methods:**

A systematic literature search was performed using PubMed, EMBASE, and Web of Science up to October 2019 to identify the eligible radical prostatectomy (RP) studies focusing on understanding the impact of age on clinicopathological features and oncological prognosis in patients with localized PCa in PSA era. Meta‐analyses were conducted using available hazard ratios (HRs) from both univariate and multivariate analyses.

**Results:**

Twenty‐six studies including 391 068 patients with RP treatments from the PSA era were included. Of these studies, age of 50 years old (age50) is the most commonly used cut‐off age to separate the younger patient group (including either age < 50 or age ≤ 50) from the older patient group. In these studies, the incidence of younger patients varied between 2.6% and 16.6% with a median of 8.3%. Younger patients consistently showed more favorable clinicopathological features correlated with better BCR prognosis. Meta‐analyses showed a 1.38‐fold improved BCR survival of younger patients in multivariate analysis. Among the high‐risk PCa patients, younger age was independently associated with worse oncological outcomes in multivariate analyses.

**Conclusion:**

In this study, we found younger age correlated with favorable clinicopathological characteristics and better BCR prognosis in low‐ to intermediate‐risk patients. In high‐risk group patients, younger patients often showed significantly worse oncological outcomes. Our study results suggest that age 50 could be used as a practical cut‐off age to separate younger age patients from older age PCa patients.

## Graphical abstract

Younger age correlated with favorable clinicopathological characteristics and better BCR prognosis in low‐ to intermediate‐risk patients. In high‐risk group, younger patients often showed significantly worse oncological outcomes. Age 50 can be used as a practical cut‐off age to separate younger age patients from older age patients.

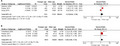

## 1 INTRODUCTION

Prostate cancer (PCa) is the most commonly diagnosed noncutaneous malignancy in men and the second leading cause of cancer‐related deaths among men in the United States and Europe.^1^ PCa has been considered a disease of older men because the incidence of PCa diagnosis increases rapidly after age 55 years and with a peak age around age 65 years at an approximate 80% rate.^2^ However, autopsy study demonstrated that the prevalence of PCa between age 40 and 50 years was roughly 20%‐30% in western cohorts,^3^, ^4^ and it was reported that the incidence of PCa between ages 15 and 40 years has increased steadily with a global averaging of 2% per year since 1990.^5^ With the widespread use of prostate‐specific antigen (PSA) screening, the incidence of PCa patients younger than 50 years increased by 5‐fold during the last decades.^6^ Considering longer life expectancy and fewer comorbidities, radical prostatectomy (RP) still remains as the standard treatment option for younger men with localized PCa. Currently, without the consensus of a standardized cut‐off age separating younger and older age patients, a wide range of ages including age 45, 50, 55 or 60 years old, has been used as a cut‐off in different reported studies.

The natural history of PCa and the impact of younger age on PCa are still poorly understood. It was speculated that the early onset PCa might be biologically different from PCa in older men and represented a larger proportion of hereditary disease.^7^ Thus far, only limited biologic and genetic evidence have been linked directly to PCa initiation and progression in younger age patients. Previous studies conducted in the pre‐PSA era suggested younger patients at ages between 15 and 40 years old showed six times more likely to have distant disease at diagnosis than older men,^5^ and tumors in patients aged < 50 years were reported to present with more aggressive characteristics than those found in older patients.^8^, ^9^, ^10^, ^11^ In contrast, more contemporary analyses described that younger PCa patients who underwent RP showed more favorable clinicopathological features and better oncological outcomes than older men in the PSA era,^12^, ^13^, ^14^, ^15^, ^16^, ^17^ although a higher prostate cancer‐specific mortality (PCSM) of younger men was found in the high‐risk group.^18^, ^19^ Literature reviews on PCa in younger age patients by Hussein et al^20^ in 2014 and by Salinas et al^21^ in 2015 have stated the potential reasons for the increasing incidence in younger men, the association between younger age and early onset or familial PCa with more aggressive disease, and the genetic risk of PCa between young‐age and elderly patients. Since the publication of these reviews, more work regarding clinicopathologic characteristics of younger patients and the impact of early screening prognosis of PCa after RP have been reported.^22^, ^23^, ^24^, ^25^, ^26^, ^27^, ^28^, ^29^, ^30^, ^31^, ^32^, ^33^, ^34^ In recent years, to avoid the overtreatment of indolent low‐risk PCa detected by PSA screening, active surveillance (AS) treatment option has been increasingly adopted.^35^ When the safety and feasibility of AS on younger patients were investigated by Salari et al,^36^ they reported that AS outcome for men aged ≤ 60 years was similar to those patients at an age older than 60 years.

In our study, systematic review and meta‐analysis were performed to analyze the differences of clinicopathological characteristics and BCR prognosis between younger and older men treated with RP to further understand the nature and impact of younger age on PCa progression.

## 2 MATERIALS AND METHODS

### 2.1 Search strategy

The literature search was conducted independently by two authors (YZ and SW) up to October 2019 to identify studies investigating clinicopathological features and/or oncological outcomes of patients underwent RP in younger only or younger and older men with PCa. PubMed, EMBASE, and Web of Science were used as search engines. The following keywords were used in our search strategy: “age” and/or “younger” and “prostate cancer” and “radical prostatectomy.” Additionally, we performed a manual search from references of included articles to retrieve other applicable studies. English‐language restriction was applied without restriction on publication date in the retrieval strategy.

### 2.2 Selection of studies

Studies were deemed eligible if they included PCa patients underwent RP in the PSA era (The FDA approved the PSA test in 1986 for the monitoring of prostate cancer progression. In 1994, the agency granted approval for the use of the PSA test in conjunction with a digital rectal examination to test asymptomatic men for prostate cancer.)^37^ (P) who had categories of younger age (I) and older age (C) and to compare clinicopathological features and oncological prognosis (including BCR, PCSM, OS, and other cause mortality (OCM)) (O). The exclusion criteria were as follows: (a) Case reports, non‐published materials, editorials, reviews, commentaries, and conference abstracts; and (b) Patients who only received radiotherapy, hormone therapy or other therapy. The most recent report or study with more complete information was used in this analysis when the same population was published in multiple studies. Only studies providing hazard ratios (HRs) from univariate and multivariate analyses with their corresponding 95% confidence intervals (CIs) were chosen for our meta‐analysis.

### 2.3 Data extraction

The relevant data of included studies were extracted with a well‐designed form. For each selected study, the following items were recorded: first author's name, year of publication, country of origin, recruitment period, categories of age, sample sizes, percentage of younger patients (including either age < 50 or age ≤ 50), race, PSA, clinical T stage, pathology T stage, D’Amico risk, biopsy Gleason score (Bx‐GS) and RP GS, rate of positive surgical margin (PSM), extraprostatic extension (EPE), seminal vesicle invasion (SVI) and lymph node invasion (LNI), Body‐mass index (BMI), follow‐up time (month), information on BCR, PCSM, OS, OCM and metastasis (Mets), Kaplan‐Meier curve (K‐M) as well as HR, 95% CI, *P*‐value from univariate and multivariate analyses and covariates of multivariate analysis.

### 2.4 Quality assessments

The quality of the study was determined by a 9‐score system named Newcastle‐Ottawa Scale (NOS) for cohort studies.^38^ Studies estimated with this system were considered of high quality if achieved a score of seven or more.

### 2.5 Study outcomes and statistical analysis

The primary outcome in this study was BCR. The secondary outcomes were Mets, PCSM, OCM, and OS.

The relevant data were analyzed by the Review Manager Version 5.4 software (Review Manager, Version 5.4 for Windows, The Cochrane Collaboration) and STATA 14.0 (StataCorp). Heterogeneity was appraised using Cochran's *Q* test (reported *P*‐value) and I statistic. A *P*‐value of less than .1 means the presence of heterogeneity when the *Q* test was performed. An *I* ^2^‐value of more than 50% was considered an indication for moderate to serious heterogeneity. The fixed‐effect model was used when heterogeneity was not significant with a *P* value > .10; otherwise, the random‐effect was applied. All results in this study were considered significant with a two‐sided *P* value < .05. The publication bias among included studies was assessed through the inverted funnel plot visual inspection. HRs with 95% CIs from each study were used to calculate the pooled HRs.

## 3 RESULTS

### 3.1 Literature search

**FIGURE 1:**
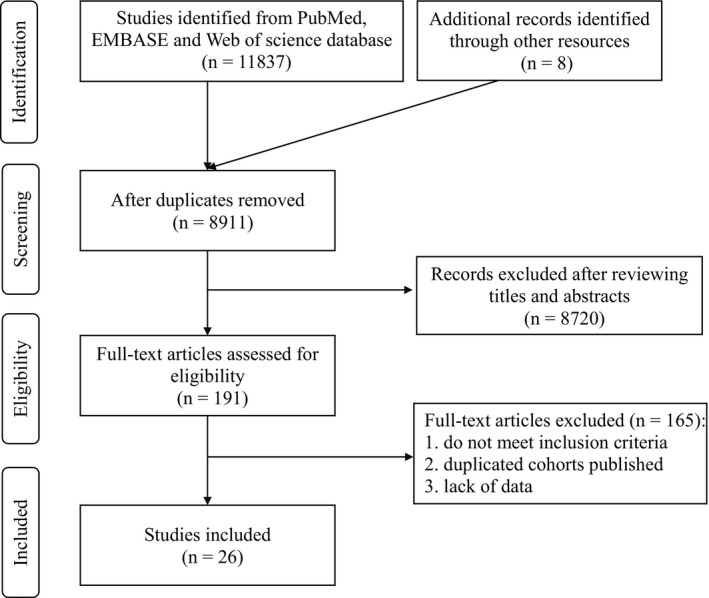
Procedure of literature search and selection of included studies

The whole process of the literature search is presented in Figure 1. The total of 11 837 studies were identified from PubMed, EMBASE, Web of science database or additional records through other resources, and 8911 studies remained after removing the duplicates. After excluding case reports, non‐published materials, editorials, reviews, commentaries, conference abstracts, and animal experiments, as well as irrelevant‐topic studies with the first browse of title and abstract, the full‐text of 191 papers, were read in detail to determine the eligibility. Finally, a total of 26 studies published from 2000 to 2019 with 391 068 patients in PSA era were included in the current study (Table 1, Stable 1),^12^, ^15^, ^18^, ^19^, ^22^, ^23^, ^24^, ^25^, ^26^, ^27^, ^28^, ^29^, ^30^, ^31^, ^32^, ^33^, ^34^, ^39^, ^40^, ^41^, ^42^, ^43^, ^44^, ^45^, ^46^, ^47^ and the majority of these studies were of relatively high quality with seven or more stars according to the NOS assessment (Table 1). Seven out of the 26 studies which provided the data on comparing clinicopathological characteristics between younger (age ≤ 50) and older (age > 50) patients were suitable for odds ratio (OR) meta‐analyses (Table 2).^22^, ^23^, ^24^, ^31^, ^39^, ^46^, ^47^ Furthermore, seven out of the 26 studies including 45 896 patients were with hazard ratio (HR) and 95% CI information for BCR prognosis and found to meet the inclusion criteria for meta‐analysis. (Tables 3 and 4).^12^, ^15^, ^23^, ^25^, ^33^, ^39^, ^44^

**TABLE 1:** Main characteristics of the included studies

Reference	Year	Country	Period	Cohort	Age group	RP cases	%age 50	Race^b^	FH	NOS
Chung [22]	2019	Korea	2001‐2017	Consecutive	<50/≥50	622 (75/547)	11.7	A	YES	9
Song [23]	2019	Korea	2006‐2015	Consecutive	≤50/>50	2057 (169/1888)	8.2	A	n/a	9
Macneil [24]	2019	Australia	2011‐2017	Consecutive	≤50/>50	11 551 (545/11006)	4.7	n/a	n/a	7
Tilki [25]	2019	Germany	2006‐2014	Consecutive	≤45/45‐65/>65	16 049 (119/8167/7763)	—	n/a	n/a	7
Pompe [26]	2018	Germany	2004‐2013	Consecutive	≤50/>50	156 719 (12956/143763)	8.3	W/B/H/O^a^	n/a	7
Tan [27]	2018	Australia	2004‐2014	Consecutive	35‐44/45‐54/55‐64/65‐74	14 324 (109/1998/6991/5226)	—	n/a	n/a	7
Sheng [28]	2018	Germany	2004‐2014	Only cT3&cT4	<50/≥50	2066 (1022/1044)	High‐risk	W/B/O^a^	n/a	7
Gielchinsky [29]	2018	Australia	1994‐2017	Only < 50	<50	339	—	n/a	YES	7
Prendeville [30]	2017	Canada	2001‐2015	Only < 50	<50	171	—	W/B/A/O^a^	YES	7
Samadi [31]	2017	USA	2002‐2012	Consecutive	≤50/>50	2495 (271/2224)	10.9	W/B/O^a^	YES^a^	8
Da Cruz [32]	2017	Brazil	1998‐2009	Matched	<55/56‐65/>65	645 (215/215/215)	—	n/a	n/a	8
Kinnear [33]	2016	Australia	1998‐2012	Consecutive	≤50/50‐70/≥70	7018 (182/3376/3460)	2.6	n/a	YES^a^	8
Dantanarayana [34]	2015	Australia	2006‐2014	Consecutive	≤55/>55	723 (110/613)	—	n/a	n/a	6
Becker [39]	2014	Canada	1992‐2011	Consecutive	<50/≥50	13 268 (443/12825)	3.3	n/a	n/a	8
Briganti [18]	2013	Italy	1987‐2010	Only high‐risk	≤59/60‐64/65‐69/≥70	3832 (973/922/1156/777)	—	n/a	n/a	8
Parker [12]	2011	USA	1989‐2009	Consecutive	<50/50‐59/60‐69/≥70	5195 (482/1896/3563/899)	9.3	W/B/O^a^	YES^a^	9
Hong [40]	2011	Korea	2003‐2008	Consecutive	<60/≥60	743 (126/617)	—	A	n/a	8
Lin [19]	2009	USA	1988‐2003	Consecutive	35‐44/45‐54/55‐64/65‐74	106 877 (881/14590/46154/45252)	—	W/B/O^a^	n/a	8
Sun [41]	2009	USA	1988‐2008	Consecutive	<60/60‐70/>70	3860 (1370/1829/661)	—	B/O^a^	n/a	8
Poulakis [42]	2007	Germany	2000‐2004	Consecutive	≤59/≥71	204 (132/72)	—	n/a	n/a	7
Siddiqui [43]	2006	USA	1987‐1995	Consecutive	<55/55‐59/60‐64/65‐69/≥70	5509 (369/640/1252/1721/1527)	—	n/a	n/a	8
Antunes [44]	2006	Brazil	1991‐2000	Consecutive	40‐49/50‐59/60‐69/70‐83	556 (23/150/268/115)	4.1	n/a	n/a	9
Rosser [45]	2006	USA	1988‐1998	Only ≤ 60	≤60	291	—	W/B/H/A^a^	n/a	7
Twiss [46]	2005	USA	2000‐2003	Consecutive	<50/≥50	790 (66/724)	8.4	n/a	n/a	7
Freedland [15]	2004	USA	1988‐2002	Consecutive	≤50/51‐60/61‐70/>71	1753 (88/473/990/202)	5.0	n/a	n/a	8
Smith [47]	2000	USA	1988‐1997	Consecutive	≤50/50‐69	477 (79/398)	16.6	W/B^a^	n/a	8

^a^ W; White, B: Black; H: Hispanic; A: Asian; O: unclassified; FH: Family history.

^b^ With statistical significance.

**TABLE 2:** Meta‐analyses of clinicopathological features by age groups (>50 vs ≤ 50) in prostate cancer

Variables^a^	Studies (N)	*I* ^2^ (%)	*P*_heterogeneity_	OR	95%CI	*P*	Effects model	References
PSA (≥10 vs <10)	3	0	0.450	1.59	1.32‐1.92	<.001	Fixed	[23, 39, 47]
cT stage (≥T2 vs T1)	6	75	0.001	0.99	0.71‐1.38	.940	Random	[22, 23, 31, 39, 46, 47]
BxGS (≥7 vs <7)	5	0	0.760	1.42	1.25‐1.62	<.001	Fixed	[22, 23, 31, 39, 46]
Damico (M + H vs Low)	3	0	0.640	1.69	1.46‐1.95	<.001	Fixed	[23, 31, 39]
RPGS (≥7 vs <7)	6	84	<0.001	1.17	1.23‐2.39	.002	Random	[22, 23, 24, 31, 39, 46]
pT stage (T3 vs T2)	7	74	<0.001	1.77	1.36‐2.32	<.001	Random	[22, 23, 24, 31, 39, 46, 47]
PSM (+ vs −)	6	8	0.360	1.30	1.10‐1.53	.002	Fixed	[22, 23, 31, 39, 46, 47]
SVI (+ vs −)	3	6	0.340	2.47	1.74‐3.52	<.001	Fixed	[23, 24, 47]
LNI (+ vs −)	3	83	0.003	1.42	0.65‐3.10	.380	Random	[23, 24, 39]

^a^ BxGS, biopsy Gleason score; Damico M + H, intermediate + high; RPGS, Gleason score of radical prostatectomy; PSM, positive surgical margin; SVI, seminal vesicle invasion; LNI, lymph node invasion.

**TABLE 3:** Summary of BCR‐prognostic information in the included studies

Reference	BCR			
	K‐M^a^	Univariate	Multivariate	Covariates
Chung [22]	*P* = .644 (Y vs O, similar)	n/a	n/a	n/a
Song [23]	*P* = .667 (Y vs O, similar)	YES (similar)	n/a	n/a
Tilki [25]	*P* < .05 (Multi‐Group, Y better)	YES (Y better)^b^	YES (similar)	Age/PSA/pT/GS/LNI/PSM
Samadi [31]	*P* = .63 (Y vs O, similar)	n/a	n/a	n/a
Da cruz [32]	*P* = .739 (Multi‐Group, similar)	n/a	n/a	n/a
Kinnear [33]	n/a	n/a	YES (similar to ≥ 70)	Age/bx‐GS
Becker [39]	n/a	n/a	YES (similar)	Age/PSA/pT/GS/LNI/PSM
Parker [12]	*P* < .0001 (Multi‐Group, Y better)	YES (Y better)^b^	YES (Y better)^b^	Age/PSA/pT/GS/Race/PSM
Hong [40]	*P* = .288 (Y vs O, similar)	n/a	n/a	n/a
Sun [41]	*P* < .01 (Multi‐Group, Y better)	n/a	n/a	n/a
Poulakis [42]	*P* = .042 (Y vs O, Y better)	n/a	n/a	n/a
Siddiqui [43]	n/a	YES (similar)	YES (similar)	data not shown
Antunes [44]	n/a	YES (Y better)^b^	YES (similar)	Age/PSA/cT/GS
Freedland [15]	*P* < .05 (Multi‐Group, Y better)	n/a	YES (Y better)^b^	Age/PSA/cT/GS/PSM/SVI
Smith [47]	*P* = .010 (Multi‐Group, Y better)	n/a	n/a	n/a

^a^ K‐M, Kaplan‐Meier; Y, younger group; O, older group.

^b^ With statistical significance.

**TABLE 4:** BCR‐prognostic information from univariate and multivariate analyses

References	Ref‐age	Age group	Univariate		Multivariate	
			HR (95% CI)	*P* value	HR (95% CI)	*P* value
Parker [12]	<50	50‐59.9	1.453 (1.149‐1.838)	.0018	1.324 (1.028‐1.704)	.0295
		60‐69.9	1.509 (1.203‐1.893	.0004	1.352 (1.058‐1.727)	.016
		≥70	1.717 (1.345‐2.191)	<.0001	1.519 (1.162‐1.985)	<.0001
Freedland [15]	<50	50‐59	/	/	2.22 (1.05‐4.66)	.036
		60‐69	/	/	2.05 (0.99‐4.26)	.053
		≥70	/	/	3.49 (1.56‐7.80)	.002
Antunes [44]	40‐49	50‐59	1.72 (0.53‐5.62)	.369	/	/
		60‐69	2.61 (0.83‐8.30)	.102	/	/
		70‐83	2.31 (0.70‐7.55)	.168	/	/
		(60‐83/40‐59)	1.56 (1.07‐2.27)	.022	1.17 (0.79‐1.73)	.426
Becker [39]	<50	≥50	/	/	0.9 (0.9‐1.2)	.9
Song [23]	≤50	>50	0.927 (0.656‐1.310)	.669	/	/
Kinnear [33]	≥70	≤50	/	/	0.69 (0.39‐1.24)	.22
		50‐70	/	/	0.72 (0.53‐0.97)	.03
Tilki [25]	>65	≤45	0.52 (0.29‐0.94)	.031	1.09 (0.58‐2.02)	.798
		45‐65	0.81 (0.76‐0.88)	<.001	1.01 (0.93‐1.08)	.86

### 3.2 Data analysis 

#### 3.2.1 Study characteristics

All 26 identified studies presented data on clinicopathological features and/or oncological outcomes in younger PCa patients with or without evaluating these data in older men counterpart (Table 1). These studies were conducted in the United States (9), Australia (5), Germany (4), Korea (3), Canada (2), Brazil (2), and Italy (1). Thirteen studies included cases from the year 2000 to the year 2017, and the other 13 studies included cases between the year 1987 to the year 2000 and thereafter. Twenty of the 26 studies were with consecutive retrospective cohorts which included both younger patients and older patients, three studies were only with the younger age patients, two studies were with selected high‐risk PCa patients, and one study was an age‐matched control study. All or some of the patients in these studies were treated with RP and only those treated with RP were included in our analysis. We found 15 of these 26 articles (58%) used age 50 years old (age50) as the cut‐off value. The proportion of age50 and younger group varied between 2.6% and 16.6% with a median of 8.3%.

#### 3.2.2 Clinicopathological characteristics

##### 3.2.2.1 Systematic review

Of studies with consecutive RP patients (n = 20), 11 studies evaluated PSA as a continuous value and 5 of them showed that younger patients had significantly lower PSA value. In five of the six studies which separating PSA into different categories, younger patients showed a higher frequency in the low PSA group (PSA ≤ 10 ng/mL; range from 73.2% to 89.1%). In 3 of the 10 studies evaluating cT stage, all studies showed that younger patients were with a significantly higher proportion of low cT stage (≤cT1; range from 25.0% to 68.0%), and only one study showed a significantly lower proportion of ≤ cT1 in younger patients (30.5% vs 52.1%).^22^ Younger patients showed significantly lower pT stage in 7 of 12 studies (≤pT2; range from 64.2% to 88.2%), higher proportion of BxGS ≤ 6 in 8 of 11 studies (range from 35.4% to 78.3%), RP GS ≤ 6 in 6 of 14 studies (range from 13.6% to 69.3%), and low‐risk PCa by D’Amico risk criteria in 4 of 5 studies (range from 46.7% to 59.9%). In addition, 4 of the 14 studies with PSM comparisons indicated that younger patients had a significantly lower PSM rate (range from 5.1% to 23.5%) when compared with older patients. Furthermore, three of the five studies with EPE comparisons showed a significantly lower frequency of EPE (range from 16.0% to 30.1%) and three of the seven studies have a significantly lower frequency of SVI (range from 3.6% to 8.3%) and LNI (range from 0% to 5.2%) in younger patients (Stable 1). In two of the three studies with metastasis information, one study reported a significantly better metastasis‐free survival of younger patients when compared with older patients (age < 60 vs age > 70) and another study showed that older patients had a significantly higher frequency in metastasis than younger age patients (≤50 vs >50; 2.5% vs 3.2%).

In the two selected high‐risk cohorts, one cohort provided the clinicopathological data for all the patients from the whole cohort but no data for the cases from RP cohort, therefore, no analysis was performed using this cohort.^28^ With a definition of high‐risk including PSA > 20 ng/mL or cT ≥ 3 or BxGS ≥ 8, the second cohort reported that patients ≥ 70 years old were with significantly lower PSA level and lower frequency of cT1 stage when compared with the younger patient groups.^18^ As for GS in this study, in contrast, they found that younger patients at age ≤ 59 years had a much higher frequency of BxGS ≤ 6 and a lower frequency of GS > 8 than older patients at age ≥ 70 years (47.3% vs 36.2%) and (22.2% vs 29.2%). No significant difference in PSM between age groups was found in this cohort.

##### 3.2.2.2 Meta‐analysis of clinicopathological characteristics

With available clinicopathological data comparing younger (age ≤ 50) and older (age > 50) patients, the data of PSA level, cT stage, biopsy GS, D’Amico risk group, RP GS, pT stage, PSM, SVI, and LNI were extracted from these studies, and the pooled OR and 95% CI were calculated. Of these characteristics, cT stage and LNI were with high study heterogeneity and did not show any significant impact between the younger and older age groups. All the other clinicopathological features showed significant OR differences between the younger and the older age groups, with the worse characteristics in the older group patients (Table 2).

#### 3.2.3 Oncological outcomes

##### 3.2.3.1 Systematic review

For oncological outcomes, BCR was reported in 15 of the 26 studies (Table 3). Among these, 11 studies provided Kaplan‐Meier (K‐M) curve and log‐rank test results, 5 studies were with univariate analyses, and 7 studies were with multivariate analyses. Six of the 11 studies with the K‐M results showed that younger patients had a significantly better BCR‐free survival. Three of the five studies with univariate analyses showed that younger patients had significantly better BCR‐free survival. In multivariate analysis, two of the seven studies showed significantly better survival of patients in age50 and younger group but the other five studies when investigating the BCR‐free survival, no statistical significance was identified between the youngest and older group patients.

When other oncological outcomes were examined (Stable 2 & 3), six studies evaluated the PCSM outcomes. In the two studies with consecutive cohorts, the authors found the younger patients had significantly better prognoses than the older patients,^26^, ^41^ and in one selected high‐risk cohort (high‐risk defined as PSA > 20 ng/mL or cT ≥ 3 or BxGS ≥ 8), younger patients had significantly worse prognosis in univariate analysis.^18^ Interestingly, in a study focusing on locally advanced PCa (clinical stage T3‐T4, M0), age50 and younger group patients were found to associate with worse PCSM prognosis independently (*P* = .02, HR:1.62, 95%CI (1.08‐2.44)).^28^

The OS outcome was also investigated in four studies. Two studies with consecutive cohorts showed significantly better prognosis for younger patients,^27^, ^44^ and significantly worse prognosis was found in one selected high‐risk cohort by K‐M test.^28^ In univariate analysis, one study with consecutive cohort showed that the youngest age group (age 35‐44) had a similar OS prognosis as the oldest age group (age 65‐74); however, other younger age groups (age 45‐54 and age 55‐64) had significantly better OS than the oldest age group (age 65‐74) which was confirmed by multivariate analysis.^27^ In another study with multivariate analysis, when compared to the youngest age group (age 35‐44), the older age groups (age 45‐54, age 55‐64 and age 65‐74) showed significantly worse OS in lower GS (≤7) patients, but showed significantly better OS in higher GS (≥8) patients except for the cases in the oldest age group (age 65‐74) which showed no significant difference when compared with younger age groups.^19^

In three studies provided OCM outcome data, two selected high‐risk studies showed a better OCM prognosis for the younger patients than the older patients in the univariate analysis which was confirmed by multivariate analysis in one of the studies.^18^, ^28^ Moreover, one study with consecutive cohort showed that older patients had a much better OCM prognosis in multivariate analysis. (*P* < .001, HR 3.02, 95% CI (2.59‐3.53)).^26^

##### 3.2.3.2 Meta‐analysis of BCR

Among the 26 studies included in our study, seven studies provided information on HR and 95% CI from either univariate or multivariate analyses on BCR survival. (Table 4) Age 50 was used as the cut‐off age to separate the younger patient group from the older patient groups in five of the seven studies which were included in further meta‐analysis, and the older patient group was either defined as age > 50 years or divided into different age subgroups in patients with age > 50 years. Five studies accounted for a total of 22 829 patients, including three studies with univariate and three studies with multivariate analyses, were used to perform the meta‐analysis. These consecutive retrospective cohort studies, with sample sizes ranged from 556 to 13 268, were published between 2004 and 2019. HR and 95% CI information from age 50 to 59‐years‐old group (if older age were divided into subgroups) were utilized for the meta‐analysis because the smallest HR with statistical significance was consistently found in patients of this age group. The mean follow‐up time in these studies ranged from 37 to 76 months.

**FIGURE 2:**
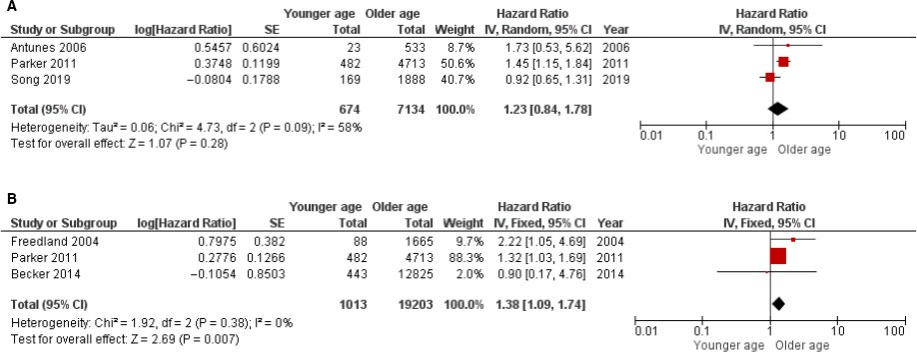
Forest plots of BCR prognosis of age. The horizontal lines correspond to the study‐specific hazard ratio and 95% confidence interval, respectively. The area of the squares reflects the study‐specific weight. The diamond represents the pooled results of hazard ratio and 95% confidence interval. (A) univariate analysis. (B) multivariate analysis

Our meta‐analysis results showed that patients at age50 or younger patients had significantly better BCR‐free survival than older patients in multivariate analysis (HR 1.38, 95% CI 1.09, 1.74; *P* = .007) (Figure 2).

#### 3.2.4 Publication bias

Through the inverted funnel plot, no publication bias was detected for any data used in meta‐analysis.

## 4 DISCUSSION

Prostate cancer is considered an aging disease of men and the characteristics of younger patients is of great interest in recent years since this entity has been increased rapidly in the PSA era. Despite a steady increase in choosing AS for the management of low‐risk PCa patients in recent years,^48^ younger men are often counseled toward active treatment because of longer life expectancy, fewer comorbidities, and the perceived likelihood of intervention required ultimately. Currently, there is an urgent need for a better understanding of the significance of younger age on PCa development and progression; therefore, helping the decision‐making process when treating younger PCa patients in the PSA era.

Since the PSA test was approved by the FDA in 1986, in general, the PSA era has been considered to start from then. However, it is difficult to define a specific time point or year as the cut‐off to guaranty the quality of the method for serum PSA screening especially between the year 1986 and 2000. In our current 26 included studies, 13 studies included cases only from the year 2000 to the year 2017, and the other 13 studies included cases between the year 1987 to the year 2000 and thereafter. Based on the fact that comparable baseline characteristics between these studies were observed, we decided to include all these studies as cases in the PSA era to provide more variables for data analysis.

In the previous studies, without any consensus or standards, a wide range of cut‐off age was used to separate the younger patients from older patients. Based on all the studies included in our analysis, we found that age 50 was the most commonly used cut‐off age for separating younger patients from older patients with a median of 8.3% incidence of younger patients in consecutive RP cohorts. Patients in the age50 and younger group consistently showed significantly more favorable clinicopathological features and better oncologic outcomes when compared with the older age patients in these consecutive cohorts. Interestingly, it had shown that the steady growth of the prostate slowly accelerated at the age of 50 years,^49^ and after the age of 50 years, men's androgen levels could drop dramatically,^50^ and the aberrance of androgen/androgen receptor (AR) axis was well‐accepted to play a critical role in the development and progression of PCa.^51^ Considering these important biologic turning points and the rapidly increased comorbidity after age 50 years, we think it is reasonable to define patients in the age ≤ 50‐years‐old group as younger patients. Our meta‐analysis results further supported the notion that age50 could be a practical and meaningful cut‐off age when studying the impact of younger age on PCa progression and considering treatment options.

In the studies with consecutive cohorts, age50 and younger group showed to associate with significantly favorable BCR prognosis independently in two studies,^12^, ^15^ and OCM prognosis in one study.^26^ As for PCSM or OS, younger patients showed better prognosis especially in the lower GS (≤7) groups; however, such results were not consistent.^19^, ^26^, ^27^ When investigating the high‐risk PCa disease, Song et al^23^ reported that patients at age ≤ 50 years showed a trend of worse BCR prognosis when compared with matched older patients. Lin et al^19^ also found that patients in the youngest age group (age 35‐44) with higher GS (≥8) had a worse OS prognosis when compared with older patients (age 45‐54 and age 55‐64). In the selected high‐risk study, Sheng et al^28^ reported that younger patients of age < 50 years showed a significantly higher PCSM in multivariate analysis. When other oncologic outcomes were analyzed, although not all the studies used age50 as age cut‐off, consistently, younger patients showed worse BCR, PCSM and OS, and PCSM was found to be a leading cause of death for younger patients without comorbidity (Table 5). These results suggested that younger patients with high‐risk PCa should be treated immediately with multi‐modality approaches to achieve the best treatment outcome.

**TABLE 5:** Selected studies discussing high‐risk PCa group after RP

References	Age group	High‐risk	Comments
Song [23]	≤50/>50	D’Amico risk group	≤50 group: trend of worse BCR‐free survival in H‐risk after matching (KM, *P* = .073)
			Comparable BCR‐free survival in L‐risk and M‐risk group
Sheng [28]	<50/≥50	Non‐metastatic cT3‐4	<50 group: significant higher PCSM (MV, *P* = .048) in LAPC patients
Gielchinsky [29]	<50	D’Amico risk group	<50 group: BCR rate at 5y in H‐risk (23.3%) lower than matched older group
Briganti [18]	≤59/60‐64/65‐69/≥70	PSA > 20/cT ≥ 3/bxGS > 7	≤59 group with no comorbidities: CSM was the leading cause of death
			OCM was the leading cause of death in all patient groups
Hong [40]	<60/≥60	D’Amico risk group	<60 group: lower BCR‐free survival in H‐risk(KM, *P* = .017)
			Independent worse BCR‐free survival in H‐risk(MV, *P* = .001)
Lin [19]	35‐44/45‐54/55‐64/65‐74	pT ≥ 3&GS > 7	35‐44 group: with highest risk of OS and PCSM (MV, *P* < .05)
Siddiqui [43]	<55/55‐59/60‐64/65‐69/≥70	GPSM (9‐16)	<55 group: significant lower sPFS comparing other groups (*P* = .041)

GPSM, mayo clinic grade based on GS; H, High risk; L, Low risk; LAPC, locally advanced prostate cancer; M, intermedium risk; MV, multivariate analysis; PSA, SVI, PSM; sPFS, systemic progression‐free survival; UV, univariate analysis.

Previously, family history and race were found to be correlated with early onset PCa among younger patients in previous studies.^52^, ^53^ In our study, three of six studies (50%) reported a significantly higher frequency of patients with family history in the age50 and younger group (range 25.8%‐36.9%). African American (AA) race was reported in nine studies at the frequency ranging from 9% to 37% and was associated with a significantly higher incidence in younger patients (Table 1). Previously, it was reported that AA race was 1.75 times more likely to be diagnosed with PCa and 2.4 times more likely to die from the diseases,^54^ hence, the higher incidence of AA could be the result of increased high‐risk PCa among younger patients.

In our study, using studies provided data on univariate or multivariate analyses,^12^, ^15^, ^23^, ^39^, ^44^ we conducted a meta‐analysis on BCR prognosis. Since the age 50‐59 years group always showed the smallest HR with statistical significance, it was reasonable to pool HR from this age group to provide an estimation for the least risk of older patients when compared to younger patients. The pooled HR (HR, 1.38; 95%CI 1.09‐1.74; *P* = .007) from multivariate analyses suggested older age could be an independent predictor for poor BCR prognosis however the risk was limited.

We also analyzed the potential association of any clinicopathological characteristics between the younger and older age groups, and the meta‐analysis results demonstrated that younger PCa patients were with significantly lower PSA, BxGS, proportion of high‐risk D’Amico disease, RP GS, pT stage, PSM, and SVI which indicated an overall favorable clinicopathological characteristic comparing to older patients.

With the rapid increase in the incidence of younger PCa patients in the last decades, more studies were focused on understanding the impact of aging on PCa. Since the publications of the reviews on this topic in 2014 by Hussein et al^20^ and in 2015 by Salinas et al^21^, 13 more studies discussing the impact of younger age on the PCa clinicopathological features or outcome prognosis in younger patients were published. In these studies, 2 of 2 studies with multivariate analyses showed similar BCR‐free survival between younger and older patient groups,^25^, ^33^ but 2 of the 5 articles^12^, ^15^ showed younger age was an independent predictor for better BCR prognosis in the studies published before 2015.

With recent efforts to identify biomarkers for sorting indolent PCa from fatal PCa, emerging genetic alternations associated with early onset PCa in younger patients were found including mutations in CTLA4, IDO1/TDO2, HOXB13, BRCA2, and TMPRSS2‐ERG fusion driven by androgen receptor.^55^, ^56^, ^57^, ^58^, ^59^, ^60^ (Stable 4) More comprehensive studies on separating high‐risk disease from low‐ to intermediate‐risk disease in younger age patients are warranted urgently.

AS has been considered a viable option for patients with low‐risk PCa. Mahran et al^35^ found that younger patients (age < 65.8) on AS have both lower risk of GS upgrading and biopsy progression when compared to older patients (age > 65.8) in their meta‐analysis study. Most recently, Salari et al^36^ reported that AS could serve as a viable option for men at age ≤ 60 years with low volume and low‐risk PCa. From our meta‐analysis, we found that younger patients at age ≤ 50 years carried a 1.4‐fold better BCR prognosis when compared with older patients after RP. Our findings may help physicians to consider AS for younger patients with age ≤ 50 years who meet the AS criteria.

Several limitations should be acknowledged in this study. First, owing to obvious differences in enrolled cohorts and different categorizations for older age patient groups, results of meta‐analysis may require cautious interpretation. Second, meta‐analysis on other prognoses including PCSM and OS were not performed due to the lack of sufficient data available.

## 5 CONCLUSION

To our knowledge, this is the first meta‐analysis performed focusing on understanding the impact of younger age on PCa progression and BCR outcome. Our results indicate that younger age is significantly associated with favorable clinicopathological characteristics and with better BCR‐free survival in patients with low‐ to intermediate‐risk PCa who underwent RP. We also found that age 50 years could be considered the practical cut‐off age to separate younger age PCa patients from older age PCa patients. Future studies on evaluating biologic subgroup and genetic markers will help to identify younger PCa patients who will benefit the most from the targeted treatment option.

## CONFLICT OF INTEREST

The authors declare no conflict of interest.

## AUTHORS CONTRIBUTION

Wu CL and Blute ML were involved in protocol development. Wu CL, Zheng Y, Lin SX, and Wu S and were involved in the project development. Zheng Y and Wu S were involved in the data collection. Zheng Y, Lin SX, Wu S, Dahl DM, Blute ML, Zhong WD, Zhou X, and Wu CL were involved in data analysis and interpretation. Zheng Y, Lin SX, Wu S, and Wu CL wrote the manuscript. Dahl DM, Blute ML, Zhong WD, and Zhou X edited the manuscript.

## ETHICAL APPROVAL

Ethical approval is not required for this kind of review study.

## Supporting information

Table S1

Table S2

Table S3

Table S4

## Data Availability

The authors confirm that the data supporting the findings of this study are available within the article and its supplementary materials.
