# Supplementary material for: Clinicopathological characteristics of localized prostate cancer in younger men aged ≤ 50 years treated with radical prostatectomy in the PSA era: A systematic review and meta‐analysis
Source: Cancer Med. 2020 Jul 22;9(18):6473–84. doi: 10.1002/cam4.3320 (PMC7520296; doi:10.1002/cam4.3320)
Supplement: Supplementary file 1 — Table S1 [file CAM4-9-6473-s001.docx]

Stable 1-1. Clinicopathological characteristics of the included studies: (% in age group; * showing significant difference)

| **Reference** | **PSA (ng/ml) or PSA Group(%)** | **cT Stage(%)** | **pT Stage(%)** | **D’Amico Risk (L/M/H )^$^(%)** |
| --- | --- | --- | --- | --- |
| Chung [22] | (12.1±1.6)/(11.0±0.5) (mean) | T1c/T2/≥T3 | T2/≥T3 | n/a |
|  |  | (30.5/56.2/13.3)//(52.1/41.2/6.5)* | (69.3/30.7)//(68.0/32.0) |  |
| Song [23] | <10/10-20/>20 | T1c/T2 | n/a | (46.7/36.7/16.6)//(35.2/36.1/28.7)* |
|  | (70.4/20.7/8.9)//(63.3/22.7/13.9) | (68.0/32.0)//(58.5/41.5)* |  |  |
| Macneil [24] | n/a | n/a | n/a | n/a |
| Tilki [25] | 6.2(5.2)/10.1(6.8)/10.2(7.0)*(mean(mdn)) | n/a | T2/≥T3(88.2/11.8)//  （70.4/29.6)//(61/39)* | (56.6/34.5/8.9)//(36.9/46.2/16.9)  //(26.9/49.8/23.3)* |
| Pompe [26] | 5.2(3.8-8)/6.4(4.8-10.1)*^&^(whole cohort) | T1/T2/≥T3 (n/a) | n/a | n/a |
| Tan [27] | 6.2 (2.2-14)/6.3 (0.3-75)/ | T1-T2/≥T3(82.6/17.4)// | n/a | n/a |
|  | 6.6 (0.2-95)/6.4 (0.4-96) ^&^ | (75.6/23.4)//(68.5/30.5)//(61.4/37.5)* |  |  |
| Sheng [28] | ≤10/10.1-20/>20  (69.2/18.5/12.3) (whole RP cohort) | T3/T4  (90.5/9.5) (whole RP cohort) | n/a | n/a |
| Gielchinsky [29] | n/a | n/a | T2/T3:Y(75.3/24.7) | Y(38.0/50.5/11.5) |
| Prendeville [30] | 5.14(0.01-284)(mdn(range) | T1c/T2/T3: Y(68/28/2.0) | T2/≥T3:Y(80/20) | n/a |
| Samadi [31] | 5.9±4.6/6.2±4.7(mean) | T1c/≥T2  (84.2/15.8)//(85/15) | T2/≥T3  (80/20)//(77/23) | (59.0/32.1/8.9)//(48.0/41.1/10.9)* |
| Da Cruz [32] | 6.59±5.02/6.75±4.43/7.04±4.08(mean) | T1c/T2/T3(66.5/31.2/2.3)//  (64.6/33.1/2.3)//(58.9/38.8/2.3) | n/a | n/a |
| Kinnear [33] | 3.1(7.7)/5.1(9.2)/8.2(15.5)*^&^ (whole cohort) | T1/T2/T3/T4(n/a) | n/a | n/a |
| Dantanarayana [34] | ≤4/4.1-10/10.1-20/>20.1  (32.7/56.4/6.4/3.6)//(17.9/65.6/14.7/1.3)* | T2/≥T3  (65.5/34.5)//(59.3/40.7) | T2/≥T3  (65.5/34.5)//(59.5/40.5) | n/a |
| Becker [39] | <4/4-10/10-20/>20  (20.8/61.6/10.5/7.1)//(13.4/61.0/19.1/6.5)* | T1c/T2/T3  (82.1/16.5/1.4)//(78.3/20.0/1.7) | T2/≥T3  (84.2/15.8)//(68.4/31.6)* | (49.3/36.4/14.2)//(34.9/46.9/18.2)* |
| Briganti [18] | 8.2(4.6–24.0)/8.8(4.6–25.0)/  8.1(4.5–23.3)/6.8(4.4-21.9)*^&^ | T1/T2/T3(29.8/32.9/37.3)//(29.6/33.6/36.8)  //(31.3/35.7/33.0)//(22.5/41.6/35.9)* | T2/≥T3(32.6/67.4)//(32.2/67.8)  //(32.4/67.5)//(33.2/66.7) | n/a |
| Parker [12] | <4/4-9/10-19/≥20  n/a | T1/T2/≥T3  (n/a) | T2/≥T3(71.5/28.5)//(63/37)  //(63/37)//(57/43)* | n/a |
| Hong [40] | 11.1/11.9(mean) | T1c/T2  (63.5/26.5)//(56.7/43.3) | n/a | (41.3/35.7/23)//(36.5/34.8/28.7) |
| Lin [19] | n/a | n/a | T2/T3/T4(n/a) | n/a |
| Sun [41] | <10/10-20/>20(79.5/14.4/6.1)// | n/a | T2/≥T3(64.2/35.8)// | n/a |
|  | (73.2/16.6/10.2)//(70.5/19.4/10.1)* |  | (58/42)//(53.7/46.3)* |  |
| Poulakis [42] | 9.1±7.1/13.5±6.4*(mean) | T1c/T2  (25/75)//(12/88)* | T2/T3  (68/32)//(55/45)* | n/a |
| Siddiqui [43] | 6.5(4.2-11.1)/6.5(4.3-11.4)/7.5(4.8-12.8)  /8.1(5.2-14.3)/8.7(5.3-15.8)*^&^ | T1/T2/≥T3(20.3/68.3/11.4)//  (24.5/64.1/11.4)//(23/65.5/11.5)// | T2/≥T3(65.2/25.3)//  (64.6/35.4)//(59.9/40.1)// | n/a |
|  |  | (23.0/66.1/10.9)//(19.9/69.9/10.2) | (56.2/43.8)//(56/44)* |  |
| Antunes [44] | <4/4.1-10/10.1-20/>20 | T1c/≥T2 | T2/T3 | n/a |
|  | (17.4/60.9/21.7/0)//(13.1/62.3/20.7/4.7)// | (52.2/47.8)//(58.7/41.3)// | (82.6/17.4)//(80.7/19.3)// |  |
|  | (7.8/53/28.7/10.4)//(8.7/57/31.3/13)* | (44/56)//(33.9/66.1)* | (73.1/26.9)//(70.4/29.6) |  |
| Rosser [45] | <4/4-10/10.1-20>20:Y^^^(19/63/14/4) | T1/T2/T3:Y(26.0/70.0/4.0) | T2/≥T3 :Y(80/20) | n/a |
| Twiss [46] | 6.3±0.6/6.5±0.2(mean) | T1c/T2  (86.2/13.8)//(85.8/14.2) | T2/T3  (86.4/13.6)//(81.7/18.3) | n/a |
| Freedland [15] | 9.8±9.0/9.1±7.8/10±10.9  /12.6±17.0(mean) | T1-T2a/≥T2b(76/24)//(76/24)  //(75/25)//(63/37) | n/a | n/a |
| Smith [47] | ≤4/4.1-10/>10  (40/38.7/21.3)//(23.9/48.6/27.5)* | T1/T2  (40.5/59.5)//(40.7/59.3) | T2/≥T3  (64.9/35.1)//(47.7/52.3)* | n/a |
| ^ Y: younger group; **^$^** L: low risk, M: medium risk; H: high risk; &: Median(IQR); | | | | |
|  |  |  |  |  |

Continued 1-2: (% in age group; * showing significant difference)

| **Reference** | **Bx GS(%)** | **RP GS(%)** | **PSM(%)** | **EPE(%)** | **SVI(%)** | **LNI(%)** |
| --- | --- | --- | --- | --- | --- | --- |
| Chung [22] | ≤6/7/≥8  (57.3/32.1/10.6)//(42.1/40.2/17.7)* | ≤6/7/≥8  (37.3/49.4/13.3)//(26.0/60.1/13.9) | 20.0/27.6 | n/a | n/a | 6.7/5.9 |
| Song [23] | <7/7/>7  (55.6/34.9/9.5)//(45.7/38.1/16.2)* | <7/7/>7  (21.3/69.2/9.5)//(11.1/76.7/12.2)* | 21.9/28.0 | 16.0/30.9* | 3.6/9.0* | 1.8/1.9 |
| Macneil [24] | n/a | ISUP  (13.6/63.9/16.0/2.8/3.9)//n/a* | n/a | 28.9//n/a* | 3.9//n/a* | 5.2//n/a* |
| Tilki [25] | ISUP(62.0/25.7/4.4/3.5/4.4)//  (47.4/29.6/11.6/6.9/4.6)//(36.4/29.9/15.8/10.2/7.7)* | ISUP(40.7/51.7/4.2/0.0/3.4)//  (19.2/59.9/15.3/0.7/5)//(12.5/58.3/20.8/1.2/7.2)* | 5.1/14.8/18.6* | n/a | n/a | 2.5/7.7/9.6* |
| Pompe [26] | ≤3+3/3+4/4+3/8-10(n/a) | n/a | n/a | n/a | n/a | n/a |
| Tan [27] | 6/7/8-10(46.8/45.9/1.8)// (35.4/51.4/6.5)  //(27.4/53/9.7)//(18.5/6.4/15.7)* | n/a | n/a | n/a | n/a | n/a |
| Sheng [28]  . | ≤6/7/8-10  (16.6/65.6/17.8) (whole RP cohort) | n/a | n/a | n/a | n/a | n/a |
| Gielchinsky [29] | n/a | n/a | Y(20.8) | n/a | Y(22.6) | Y(14.6) |
| Prendeville [30] | ISUP:Y(47.0/32.0/8.0/2.0/0.5) | ISUP:Y(42/45/10.5/0.5/1.0) | Y(19) | n/a | Y(4) | Y(2) |
| Samadi [31] | ≤6/(3+4)/(4+3)/≥8  (61.6/24.3/7.4/6.7)//(52.5/29.0/10.0/9.5)* | ≤6/(3+4)/(4+3)/≥8  (28.8/54.2/11.5/5.5)//(22.1/56.8/14.9/6.2) | 20.7/20.1 | n/a | n/a | n/a |
| Da Cruz [32] | ISUP(40.2/13/21.3/21.3/4.2)//  (40.2/13/21.3/21.3/4.2)//( 40.2/13/21.3/21.3/4.2) | n/a | n/a | 22.9/22.1/26.8 | 10.3/9.8/8.5 | 0.5/0.5/0.5 |
| Kinnear [33] | <7/(3+4)/(4+3)/>7(n/a) | n/a | n/a | n/a | n/a | n/a |
| Dantanarayana [34] | n/a | ISUP  (23.6/51.8/17.3/2.7/4.5)//(15.7/53.2/20.9/2.1/8.2) | 24.5/25.4 | n/a | 7.3/7.7 | 3.6/2.2 |
| Becker [39] | ≤6/(3+4)/(4+3)/≥8  (61.6/26.3/6.5/5.6)//(54/26.7/10.8/8.5)* | ≤6/(3+4)/(4+3)/≥8  (33.1/55.9/8.7/2.3)//(28.7/53.3/13.7/4.3)* | 11.5/16.8* | n/a | n/a | 4.5/5.1 |
| Briganti [18] | ≤6/7/8-10(47.3/17.6/35.1)//(43.3/18.2/38.5)  //(43.1/14.4/42.5)//(36.2/18.9/44.9)* | ≤6/7/8-10(22.0/54.6/22.2)//(21.9/53.5/23.1)  //(20.9/54/23.8)//(21.8/48.8/29.2)* | 59.7/58.7/58/57.8 | n/a | n/a | n/a |
| Parker [12] | 2-6/7/8-10  (n/a) | 2-6/7/8-10(68/26/6)//(57/33/10)  //(54/36/10)//(50/35/15)* | 23.5/28/27.5/29* | n/a | n/a | n/a |
| Hong [40] | ≤6/7/≥8  (53.2/34.1/12.7)//(50.6/35.0/14.4) | ≤6/7/≥8  (33.4/57.9/8.7)//(24.7/64.0/11.3) | 31.7/35 | 25.4/31.8 | 8.7/8.9 | n/a |
| Lin [19] | 5-7/8-10(n/a) | n/a | n/a | n/a | n/a | n/a |
| Sun [41] | n/a | <7/7/>7(50.0/40.5/9.5)//  (42.5/44.2/13.3)//(39.6/43.1/17.3) | 34.1/33.7/36.2 | 30.1/33.8/37.9* | 8.3/11.9/13.7* | n/a |
| Poulakis [42] | n/a | n/a | 11.0/21.0* | n/a | n/a | n/a |
| Siddiqui [43] | ≤6/7/8-10(76.3/18.5/65.3)//  (75.7/20.6/3.7)//(71.9/23.9/4.2)// | ≤6/7/8-10(69.3/25.1/5.3)//  (66.3/28.4/5.3)//(65.6/28.1/6.3)// | 37.7/37.3/37.1  /39.8/39.8 | n/a | n/a | n/a |
|  | (73/21.9/5.1)//(68.5/24.3/7.2)* | (62.9/30.2/6.9)//(61.2/30.8/8.0)* |  |  |  |  |
| Antunes [44] | ≤6/7/8-10(78.3/13.0/8.7)//(87.3/10.7/2.0) | ≤6/7/8-10(65.2/17.4/17.4)//(68.7/17.3/14) | n/a | n/a | n/a | n/a |
|  | //(76.9/14.9/8.2)//(73.0/18.3/8.7)* | //(60.4/20.9/18.7)//(57.4/20.9/21.7) |  |  |  |  |
| Rosser [45] | ≤6/7/8-10:Y(52.0/38.0/10.0) | 7/8-10:Y(84.0/16.0) | Y(11.0) | Y(16) | Y(4) | Y(1) |
| Twiss [46] | ≤6/7/8-10  (74.2/19.7/6.1)//(70.9/24.6/4.4) | ≤6/7/8-10  (63.6/30.3/6.1)/(57.7/37.8/4.5) | 6.1/6.9 | n/a | n/a | n/a |
| Freedland [15] | ≤6/7/8-10(79/19/1.0)//(70/21/9.0)//  (67/24/8)//(70/21/9) | ≤6/7/8-10(50/38/12)//(57/34/9.0)//  (53/38/10)//(53/34/13) | 37/30/  33/31 | 20/23/  26/28 | 4/9/9/9 | 0/1/2/5* |
| Smith [47] | n/a | n/a | 34.7/38.9 | n/a | 9.2/12.2 | n/a |
| Bx: Biopsy; GS: Gleason score; RP: radical prostatectomy; PSM: positive surgical margin; EPE: Extraprostatic extension; SVI; seminal vesicle invasion; LNI: lymph node involvement; ISUP: ISUP grade group. | | | | | | |
|  |  |  |  |  |  |  |

Continued 1-3:

| **Reference** | **BMI (kg/m2)** | **F/U of whole cohort(M)^&^** | **BCR** | **PCSM** | **OS** | **OCM** | **Mets** | **Tx** |
| --- | --- | --- | --- | --- | --- | --- | --- | --- |
| Chung [22] | 23.6±0.4/24.2±0.1 | 78(38.0-102.0) | YES | n/a | n/a | n/a | n/a | RP |
| Song [23] | 24.34±2.49/24.3±2.59 | 53.80±29.05 | YES | n/a | n/a | n/a | n/a | RP |
| Macneil [24] | n/a | n/a | n/a | n/a | n/a | n/a | n/a | RP |
| Tilki [25] | n/a | ~60 | YES | n/a | n/a | YES | n/a | RP |
| Pompe [26] | n/a | 52(26-82) | n/a | YES | n/a | n/a | YES | RP/Other |
| Tan [27] | n/a | ~120 | n/a | YES | YES | n/a | n/a | RP |
| Sheng [28] | n/a | ~120 | n/a | YES | YES | YES | n/a | RP/Other |
| Gielchinsky [29] | n/a | 101.5(59.75-203.25) | n/a | n/a | n/a | n/a | YES | RP/Other |
| Prendeville [30] | n/a | 60.4(1.4-171.6) | n/a | n/a | n/a | n/a | n/a | RP |
| Samadi [31] | n/a | 15.4 (7.4-29.4) | YES | n/a | n/a | n/a | n/a | RALP |
| Da Cruz [32] | n/a | 80 ± 43 | YES | n/a | n/a | n/a | n/a | RP |
| Kinnear [33] | n/a | >48 | YES | n/a | n/a | n/a | n/a | RP/Other |
| Dantanarayana [34] | n/a | n/a | n/a | n/a | n/a | n/a | n/a | RP |
| Becker [39] | n/a | 37(0.1-230.2) | YES | n/a | n/a | n/a | n/a | RP |
| Briganti [18] | n/a | 72(24.8-129) | n/a | YES | n/a | YES | n/a | RP |
| Parker [12] | n/a | 76(mean) | YES | n/a | n/a | n/a | n/a | RP/Other |
| Hong [40] | 24.2/ 24.4 | 39.4 (24-67) | YES | n/a | n/a | n/a | n/a | RP |
| Lin [19] | n/a | 45 (median) | n/a | YES | YES | n/a | n/a | RP/Other |
| Sun [41] | n/a | ~144 | YES | YES | n/a | n/a | YES | RP |
| Poulakis [42] | 27±5/29±4 | >6 | YES | n/a | n/a | n/a | n/a | RP |
| Siddiqui [43] | n/a | 10.6(8.7-12.4) | YES | n/a | n/a | n/a | n/a | RRP |
| Antunes [44] | n/a | 60.5(1-131) | YES | n/a | YES | n/a | n/a | RP |
| Rosser [45] | n/a | 48.6 (median) | n/a | n/a | n/a | n/a | n/a | RP |
| Twiss [46] | n/a | ~24 | n/a | n/a | n/a | n/a | n/a | RRP |
| Freedland [15] | n/a | 51(59±43) | YES | n/a | n/a | n/a | n/a | RP |
| Smith [47] | n/a | 54 (mean) | YES | n/a | n/a | n/a | n/a | RP |
| BMI: Body mass index; **^&^**F/U: follow up; M: month; Median (range), Mean±SD, ~: about, >: over; BCR: biochemical recurrence, PCSM: prostate cancer specific morality, OS: overall survival, Mets: metastasis. Tx: treatment | | | | | | | | |
|  |  |  |  |  |  |  |  |  |
