# Supplementary material for: Clinicopathological characteristics of localized prostate cancer in younger men aged ≤ 50 years treated with radical prostatectomy in the PSA era: A systematic review and meta‐analysis
Source: Cancer Med. 2020 Jul 22;9(18):6473–84. doi: 10.1002/cam4.3320 (PMC7520296; doi:10.1002/cam4.3320)
Supplement: Supplementary file 2 — Table S2 [file CAM4-9-6473-s002.docx]

Stable 2. Other oncological outcomes in the included studies

|  |  |  |  |  |
| --- | --- | --- | --- | --- |
|  |  |  |  |  |
|  | **OS** | | | |
| **Reference** | **K-M*** | **Univariate** | **Multivariate** | **Covariates** |
| Tan [27] | P<0.05(Multi-Group, Y better) | YES (Y similar) | YES (Y similar) | Age/PSA/cT/GS/Location |
| Sheng [28]^#^ | P=0.038(Y vs O, O better) | n/a | n/a | n/a |
| Lin [19]  Antunes [44] | n/a  P=0.039(Multi-Group, Y better) | n/a  n/a | YES (Low-GS, Y better; H-GS, O better)  n/a | Age/Stage/Race/ITT/YD  n/a |
| ITT=initial treatment type; YD=year of diagnosis; Y: younger group; O: older group; OS: overall survival; GS: Gleason score; *: with statistical significance; ^#^: High-risk cohort | | | | |
|  |  |  |  |  |
|  |  |  |  |  |
|  |  |  |  |  |
|  | **PCSM** | | | |
| **Reference** | **K-M*** | **Univariate** | **Multivariate** | **Covariates** |
| Pompe [26] | P<0.01(Y vs O, Y better) | n/a | YES(Similar) | Age/pT/GS/Race/PSM/LNI |
| Tan [27] | n/a | YES(age55-64 better)* | YES(Similar) | Age/PSA/cT/GS/Location |
| Sheng [28]^#^ | P=0.046(Y vs O, O better) | n/a | YES(O better)* | Age/PSA/cT/GS/LNI/PSM |
| Briganti [18]^#^  Lin [19]  Sun [41] | n/a  n/a  P=0.007(Multi-Group, Y better) | YES(Similar)  n/a  n/a | YES(Similar)  YES(High-GS,O bettter)*  n/a | Age/CCI/pT/GS/PSM/AT  Age/Stage/Race/ITT/YD  n/a |
| AT=adjuvant treatment; ITT=initial treatment type; CCI=Charlson Comorbidity Index; YD=year of diagnosis; Y: younger group; O: older group; PCSM: prostate cancer specific mortality; GS: Gleason score; *: with statistical significance; ^#^: High-risk cohort | | | | |
|  |  |  |  |  |
|  | | | | |
|  |  |  |  |  |
|  |  |  |  |  |

|  |  |  |  |  |
| --- | --- | --- | --- | --- |
|  |  |  |  |  |
|  | **OCM** | | | |
| **Reference** | **K-M*** | **Univariate** | **Multivariate** | **Covariates** |
| Pompe [26] | n/a | n/a | YES(Y better)* | Age/pT/GS/Race/PSM/LNI |
| Sheng [28]^#^ | P<0.0001(Y vs O, Y better) | n/a | n/a | n/a |
| Briganti [18]^#^ | n/a | YES(Y better)* | YES(Y better)* | Age/CCI/pT/GS/PSM/AT |
| AT=adjuvant treatment; ITT=initial treatment type; CCI=Charlson Comorbidity Index; Y: younger group; O: older group; OCM: other cause mortality; *: with statistical significance; ^#^: High-risk cohort | | | | |
|  |  |  |  |  |
|  |  |  |  |  |
|  |  |  |  |  |
|  |  |  |  |  |
|  |  |  |  |  |
|  |  |  |  |  |
