# Supplementary material for: Clinicopathological characteristics of localized prostate cancer in younger men aged ≤ 50 years treated with radical prostatectomy in the PSA era: A systematic review and meta‐analysis
Source: Cancer Med. 2020 Jul 22;9(18):6473–84. doi: 10.1002/cam4.3320 (PMC7520296; doi:10.1002/cam4.3320)
Supplement: Supplementary file 3 — Table S3 [file CAM4-9-6473-s003.docx]

Stable 3. Univariate and multivariate analyses on other-outcomes in the available studies

|  | |  |  |  |  |  |  |  |
| --- | --- | --- | --- | --- | --- | --- | --- | --- |
|  |  | |  |  | **OS** | | | |
|  |  | |  |  | **Univariate** | | **Multivariate** | |
| **Reference** | **Cohort** | | **Ref-Age** | **Age Group** | **HR (95% CI)** | **P Value** | **HR (95% CI)** | **P Value** |
| Tan [27] | Entire | | 65-74 | 35-44 | 0.35 (0.09-1.40) | 0.13 | 0.99 (0.24-4.04) | 0.99 |
|  |  | |  | 45-54 | 0.34 (0.25-0.47) | <0.05 | 0.33 (0.20-0.56) | <0.05 |
|  |  | |  | 55-64 | 0.52 (0.45-0.62) | <0.05 | 0.54 (0.41-0.72) | <0.05 |
| Lin [19] | Gleason5-7 | | 35-44 | 45-54 | / | / | 6.41 (1.59-25.8) | <0.05 |
|  |  | |  | 55-64 | / | / | 10.1 (2.53-40.5) | <0.05 |
|  |  | |  | 65-74 | / | / | 20.5 (5.12-81.9) | <0.05 |
|  | Gleason8-10 | | 35-44 | 45-54 | / | / | 0.47 (0.27-0.84) | <0.05 |
|  |  | |  | 55-64 | / | / | 0.55 (0.32-0.96) | <0.05 |
|  |  | |  | 65-74 | / | / | 0.86 (0.49-1.50) | >0.05 |

OS: overall survival

|  |  |  |  | **PCSM** | | | |
| --- | --- | --- | --- | --- | --- | --- | --- |
|  |  |  |  | **Univariate** | | **Multivariate** | |
| **Reference** | **Cohort** | **Ref-Age** | **Age Group** | **HR (95% CI)** | **P Value** | **HR (95% CI)** | **P Value** |
| Tan [27] | Entire | 65-74 | 35-44 | 1.02 (0.14-7.40) | 0.98 | 3.39 (0.44-25.78) | 0.24 |
|  |  |  | 45-54 | 0.66 (0.38-1.15) | 0.14 | 0.58(0.20-1.68) | 0.31 |
|  |  |  | 55-64 | 0.62 (0.45-0.94) | **<0.05** | 1.04 (0.60-1.78) | 0.9 |
| Lin [19] | Gleason5-7 | 35-44 | 45-54 | / | / | 3.15 (0.44-23.7) | >0.05 |
|  |  |  | 55-64 | / | / | 2.79 (0.39-19.9) | >0.05 |
|  |  |  | 65-74 | / | / | 4.19 (0.56-29.9) | >0.05 |
|  | Gleason8-10 | 35-44 | 45-54 | / | / | 0.35 (0.19-0.65) | **<0.05** |
|  |  |  | 55-64 | / | / | 0.29 (0.16-0.52) | **<0.05** |
|  |  |  | 65-74 | / | / | 0.35 (0.19-0.63) | **<0.05** |
| Pompe [26] | Entire | ≤50 | >50 | / | / | 1.24 (0.99–1.55) | 0.07 |
| Sheng [28]^#^ | Entire | ≥50 | <50 | / | / | 1.77 (1.01-3.10) | **0.048** |
| Briganti [18]^#^ | Entire | ≤59 | 60-64 | 0.76 (0.52-1.12) | 0.2 | 0.76 (0.52-1.12) | 0.2 |
|  |  |  | 65-69 | 0.93 (0.66-1.31) | 0.7 | 0.96 (0.67-1.38) | 0.8 |
|  |  |  | ≥70 | 0.96 (0.66-1.39) | 0.8 | 0.95 (0.64-1.41) | 0.9 |

PCSM: prostate cancer specific mortality; ^#^: High-risk cohort

|  |  |  |  | **OCM** | | | |
| --- | --- | --- | --- | --- | --- | --- | --- |
|  |  |  |  | **Univariate** | | **Multivariate** | |
| **Reference** | **Cohort** | **Ref-Age** | **Age Group** | **HR (95% CI)** | **P Value** | **HR (95% CI)** | **P Value** |
| Pompe [26] | Entire | ≤50 | >50 | / | / | 3.02(2.59-3.53) | **<0.001** |
| Briganti [18]^#^ | Entire | ≤59 | 60-64 | 1.77(1.29-2.42) | **<0.001** | 1.71(1.24-2.35) | **<0.001** |
|  |  |  | 65-69 | 2.86(2.15-3.81) | **<0.001** | 2.72(2.03-3.64) | **<0.001** |
|  |  |  | ≥70 | 3.58(2.68-4.78) | **<0.001** | 3.33(2.48-4.48) | **<0.001** |

OCM: other cause mortality; ^#^: High-risk cohort
