# Supplementary material for: Clinicopathological characteristics of localized prostate cancer in younger men aged ≤ 50 years treated with radical prostatectomy in the PSA era: A systematic review and meta‐analysis
Source: Cancer Med. 2020 Jul 22;9(18):6473–84. doi: 10.1002/cam4.3320 (PMC7520296; doi:10.1002/cam4.3320)
Supplement: Supplementary file 4 — Table S4 [file CAM4-9-6473-s004.docx]

Stable 4. Emerging genetic alteration in younger age

| **Reference** | **Molecular** | **Comments** |
| --- | --- | --- |
| Ding [55] | CTLA4/ IDO1/TDO2 pathways | Significant up-regulation in PCa of the young cohort |
| Cooney [56] and Kin [57] | HOXB13 | A recurrent mutation in early-onset and/or metastatic PCa |
| Risbridge [58]and Kote-Jarai [59] | BRCA2 | Carriers have high risk of more aggressive PCa at younger age |
| Weischenfeldt [60] | TMPRSS2–ERG fusion/AR axis | Higher frequency in early-onset PCa than elderly-onset PCa |
| Cooney [56] | Mismatch repair (MMR) gene | High risk of gene mutation in younger PCa patients |

PCa: prostate cancer; AR: androgen receptor
